# Physical activity and depression symptoms in people with osteoarthritis-related pain: A cross-sectional study

**DOI:** 10.1371/journal.pgph.0003129

**Published:** 2024-07-18

**Authors:** Michaela C. Pascoe, Rhiannon K. Patten, Alexander Tacey, Mary N. Woessner, Matthew Bourke, Kim Bennell, Phong Tran, Michael J. McKenna, Vasso Apostolopoulos, Rebecca Lane, Jakub Koska, Alev Asilioglu, Jodie Sheeny, Itamar Levinger, Alexandra Parker

**Affiliations:** 1 Institute for Health and Sport (iHeS), Victoria University, Melbourne, Australia; 2 Department of Orthopaedic Surgery, Western Health, Melbourne, Australia; 3 School of Occupational Therapy, Faculty of Health Sciences, Western University, London, Canada; 4 Centre for Health Exercise and Sports Medicine, Department of Physiotherapy, Melbourne School of Health Sciences, University of Melbourne, Melbourne, Australia; 5 Australian Institute for Musculoskeletal Science (AIMSS), Victoria University, University of Melbourne and Western Health, Melbourne, Australia; 6 Orygen, The National Centre of Excellence in Youth Mental Health and Centre for Youth Mental Health, University of Melbourne, Melbourne, Australia; The Chinese University of Hong Kong Faculty of Medicine, HONG KONG

## Abstract

Osteoarthritis is a leading cause of chronic pain and is associated with high rates of depression. Physical activity reduces depression symptoms and pain levels. It remains unknown if physical activity is associated with lower symptoms of depression irrespective of pain levels in individuals with osteoarthritis. We explored whether pain mediated or moderated the relationship between levels of physical activity engagement and depression symptoms. Individuals with osteoarthritis who were waiting for an orthopaedic consultation at a public hospital in Melbourne, Australia, were recruited. Data collected on pain levels, physical activity engagement and depression symptoms. Descriptive statistics were used to summarise participant characteristics. Moderation and mediation analyses were used to establish the impact of pain on the relationship between physical activity and depression, after adjusting for demographic and joint specific characteristics. The results indicated that the inverse association between physical activity and depression depended on the level of pain, such that the association was stronger in people with greater pain. The mediation results confirm that participating in physical activity is indirectly, inversely associated with symptoms of depression through lower levels of pain. The highest levels of pain were associated with the most potential benefit in terms of reduction in symptoms of depression from engaging in physical activity. Physical activity may be particularly important to manage depression symptoms in people with greater osteoarthritis-related pain as patients with the highest pain may have the greatest benefits.

## Introduction

Osteoarthritis (OA) is a disabling and chronic condition [1]. It affects 7% of the global population, equating to more than 500 million people living with OA worldwide [2]. Many individuals living with OA experience debilitating chronic pain which decreases their mobility and negatively impacts their physical, social and mental wellbeing [3]. Individuals with OA are threefold more likely to report experiencing severe pain, and twice as likely to report experiencing high psychological distress as compared to individuals without OA [4]. Previous work shows that OA-related pain is correlated with higher levels of depression [5] and Meta-analysis shows a moderate positive correlation between pain severity and depression symptomology in individuals with OA [6]. Accordingly, many individuals with OA are also diagnosed with depression [5]. One-fifth of people with OA experience symptoms of depression, [7] ~4.5-fold higher than global estimates in the adult population of 4.4% [8]. Depression is associated with alterations to central pain processing, resulting in increased pain sensitivity and there is evidence suggesting that persistent pain is more likely to lead to depression as individuals with more severe and enduring pain are at a greater risk for more severe depression and higher suicidal ideation [9].

Physical activity, defined as any bodily movement produced by skeletal muscles that results in energy expenditure [10, 11], is an important mental health determinant in people with OA [6]. Physical activity interventions are effective at decreasing symptoms of depression in people affected by chronic pain and arthritis [12, 13]. Patients with knee OA who are inactive have higher depressive symptoms compared to individuals who are physically active [6, 14, 15]. Furthermore, physical activity is effective at reducing pain in individuals with hip [16] and knee OA [17, 18], for up to 12 months post intervention [19]. Despite the significant benefits of being physically active, individuals living with knee and hip OA commonly report that pain levels and fear of movement present significant barriers to physical activity engagement, with individuals reportedly avoiding physical activities that they are physically capable of undertaking [20–22].

However, little is known about the mediating role pain plays in the relationship between physical activity and depression in this population. It is valuable to explore if physical activity levels are differentially associated with depression symptoms in people with low, medium and high pain levels in order to identify who might most benefit from physical activity-based interventions to help manage mood symptoms. This is particularly relevant for individuals who may experience severe pain and who are therefore less likely to engage in physical activity [20–22]. It is also valuable to explore if the relationship between physical activity and depression symptoms is mediated by impacting the experience of pain, as this can assist in determining appropriate care plans and in developing targeted approaches to support individuals with OA-related pain to engage in beneficial physical activity. Therefore, in the current study we aim to better understand the impact of pain on the relationship between physical activity and depression symptoms in people with OA. In the current study it was determined whether the moderating and mediating effect of pain had a relationship between physical activity and depression in a sample of individuals with OA who are on an orthopaedic waitlist.

## Materials and methods

### Study design

This study reports on cross-sectional data collected as part of a larger research project which is currently underway and collecting longitudinal data examining the health, functioning and mental wellbeing of individuals on the orthopaedic waitlist in order to determine their needs and develop effective interventions.

### Participants

Participants were recruited from the orthopaedic outpatient waitlist at Western Health Hospital in Melbourne, Australia. Western Health provides healthcare in one of the most socio-economically disadvantaged regions of the west of Melbourne in Victoria. This is an area that also has a high prevalence of individuals from culturally and linguistically diverse backgrounds. Recruitment began September 22nd, 2021 and was completed the 29th of August 2022. Data was accessed for research purposes on the 29th of August 2022. All individuals that were placed on the waitlist between the 1^st^ of January 2018 and the 1^st^ of June 2022 and who were 18 years of age or older were invited to participate in this project. Individuals were excluded if they had dementia or other congenital diseases that impacted cognitive functions in a way that meant they were unable to consent to taking part in the study without significant help from a carer, if they had been removed from the waiting list, if they had a specialist appointment in the next 6 weeks, or if they were booked for surgery. For the purpose of the current study, individuals were included if they had OA listed on their medical referral or if they met the three clinical criteria: 1) over the age of 45, 2) had physical activity-related joint pain and 3) had no morning joint-related stiffness, or morning stiffness that lasts no longer than 30 minutes [23].

### Procedures

The study was approved by Melbourne Health Human Research Ethics Committee (2021.055) and Victoria University Human Research Ethics Committee (mirror approval). Patients who were unable to be contacted after three attempts were recorded as non-responders. For interested individuals, a survey package was sent via email, mail or completed over the phone. The survey package included a cover letter, participant information and informed consent form and the survey measures. For individuals preferring an electronic survey, a link to a secure website where individuals could complete the survey (REDCap, a secure web application for building and managing online surveys and databases) was provided. For individuals that preferred a hard-copy, the surveys were mailed out with an addressed, pre-paid return envelope to return the survey. All written communication and questionnaires were in English. If patients were unable to understand or read English, their next of kin was contacted to assist with translation. Written informed consent was obtained prior to the beginning of the questionnaires. If consent was not obtained, participants were unable to progress to the questionnaires.

### Measures

For all individuals on the orthopaedic outpatient waitlist, data were extracted from their medical records and entered into a secure online database (REDCap). Extracted data included age, gender and location/s of affected joint/s. When an individual was classified as having multiple joints affected (i.e., knee and hip) they were not also classified as having each particular joints affected (i.e. they are not listed under knee or hip, only under multiple).

#### Depression symptoms

Depression-like symptoms were measured using the Patient Health Questionnaire (PHQ-9) [24]. The PHQ-9 is a self-report measure of depression consisting of nine items matching the Diagnostic and Statistical Manual of Mental Disorders, Fourth Edition (DSM-IV) criteria of major depression. Respondents were asked to rate each of the items on a scale of 0 to 3 on the basis of how much a symptom has bothered them over the last 2 weeks (0 = not at all, 1 = several days, 2 = more than half the days, 3 = nearly every day). The summed-item score adds up the scores from each of the items to give a total score ranging from 0 to 27. Participants who selected at least “more than half the days” on the majority of questions and show symptoms of depressed mood or anhedonia on at least half the days are considered to have a major depressive disorder. Participants who select at least “more than half the days” on 2–4 symptoms, including symptoms of depressed mood or anhedonia are considered to have another depressive disorder.

#### Physical activity

Physical activity engagement levels from the Active Australia Survey (AAS) were used to assess current leisure-time physical activity [25]. The AAS is a self-report, paper-based or online 8-item measure of activity that asks participants to report the frequency and duration of physical activity in the past week, including walking, moderate and vigorous activity. Total time in activity was calculated using the equation specified in the AAS scoring manual (walking minutes + moderate minutes + 2 x vigorous minutes). As suggested in the scoring manual, levels of individual physical activity were truncated to 840 minutes. Additionally, overall level of physical activity was truncated at three standard deviations (SD) above the mean to reduce the impact of extreme outliers on the analyses.

#### Pain

Self-reported pain intensity scores were determined according to the 4-item pain intensity measurement (P4). The P4 is a 4-item pain intensity measurement instrument. The items inquire about pain in the morning, afternoon, evening, and with activity. Each item is scored on an 11-point numeric pain scale. The anchors are ‘no pain’ (0) and ‘pain as bad as it can be’ (10). Item scores are summed to yield a total score from 0 to 40 [26].

### Data analysis

All data analyses were conducted using jamovi version 1.6 (The jamovi project, https://www.jamovi.org), and R version 4.1.2 (R Core Team, Vienna, Austria) in RStudio (RStudio Team, Boston, MA).

First, patterns of missing data were examined using the naniar package [27] and it was determined that data were missing completely at random (Little’s MCAR x^2^ (34) = 40.6, p = .201). Therefore, missing data were deleted pairwise. In total, 7.1% of participants had missing data and were removed from the analysis.

Bivariate association between study variables was estimated using correlation coefficients, t-tests, and ANOVAs. Levels of physical activity had a substantial skew (skewness = 1.60); therefore, Spearman rank order correlations were calculated for physical activity. All other correlations were calculated as Pearson correlation coefficients. To determine whether pain moderated the association between physical activity and depression, linear regression models were estimated with an interaction effect between pain and physical activity. The model included participant gender and age, and the joint affected (or multiple joints) by osteoarthritis as covariates. The residuals from the model were plotted against predicted values and indicated heteroskedastic errors. Therefore, the sandwich package [28, 29] was used to estimate robust standard errors for each outcome. To determine if pain mediated the association between physical activity and depression, a mediation model was estimated using the mediation package [30] in R. The significance of indirect effects was estimated as 95% quasi-Bayesian Monte Carlo confidence intervals based on 1,000 resamples with robust standard errors.

## Results

### Descriptive statistics

The study included a total of 552 participants of which 54.7% were women, and the average participant age was 62.6 years (SD = 10.7). The most common joint affected by OA was the knee (44.0%), followed by the hip (15.5%), shoulder (14.4%), and foot (13.0%) and 9% had multiple joints affected [which may have included the above-mentioned joints]). Overall, 33.9% of participants had moderate depression scores or higher with 18.8% reporting a major depressive disorder and 10.8% another depressive syndrome. On average, participants reported engaging in 5.19 (SD = 6.5) hours of total physical activity per week. Overall, 47.8% of participants were sufficiently active based on the Australian Physical Activity Guidelines (at least 150 minutes of MVPA or 75 minutes of vigorous intensity physical activity per week), 36.9% were insufficiently active, and 15.4% were sedentary (zero minutes of MVPA per week). On a scale of 0–40, participants reported an average level of pain of 27.70 (SD = 8.6).

### Bivariate associations

Differences in depression between men and women and by joint affected are displayed in Table 1.

**Table 1:** Differences in depression by sex and joint affected by osteoarthritis.

	Depression Symptoms		
	Mean	95%CI	p-value
Sex*			.064
Male (n = 249)	7.32	6.49, 8.15	
Female (n = 302)	8.39	7.63, 9.15	
Joint Affected			.380
Knee (n = 243)	8.24	7.41, 9.07	
Hip (n = 86)	8.20	6.71, 9.69	
Shoulder (n = 80)	7.57	5.92, 9.22	
Foot (n = 72)	7.83	6.17, 9.49	
Ankle (n = 27)	6.76	4.65, 8.87	
Multiple* (n = 23)	5.65	3.62, 7.68	
Other (n = 21)	8.11	5.15, 11.07	
Hours of total physical activity per week	5.19		
Reported pain score (0–40)	27.7		

CI = Confidence interval, n = sample size. One participant identified as non-binary.

*When an individual was classified as having multiple joints affected (i.e., knee and hip) they were not also classified as having each particular joints affected (i.e., they are not listed under knee or hip, only under multiple).

Correlations between all continuous variables are presented in Table 2. Physical activity levels were inversely correlated with both depression symptom scores (r = -0.16, p < .001) and pain (r = -0.26, p < .001). Individuals who reported doing more physical activity had fewer depression symptoms and less pain. Pain was also independently positively correlated with depression symptoms, with those reporting higher levels of pain also having more depressive symptoms.

**Table 2:** Correlation coefficients between study variables.

	Physical Activity	Depression Symptoms	Age (years)	Pain Levels
Physical Activity	-			
Depression Symptoms	-0.156*	-		
Age (years)	-0.233*	-0.077	-	
Pain Levels	-0.255*	0.439*	0.071	-

* < .001

### Moderation analysis

**Fig 1:**
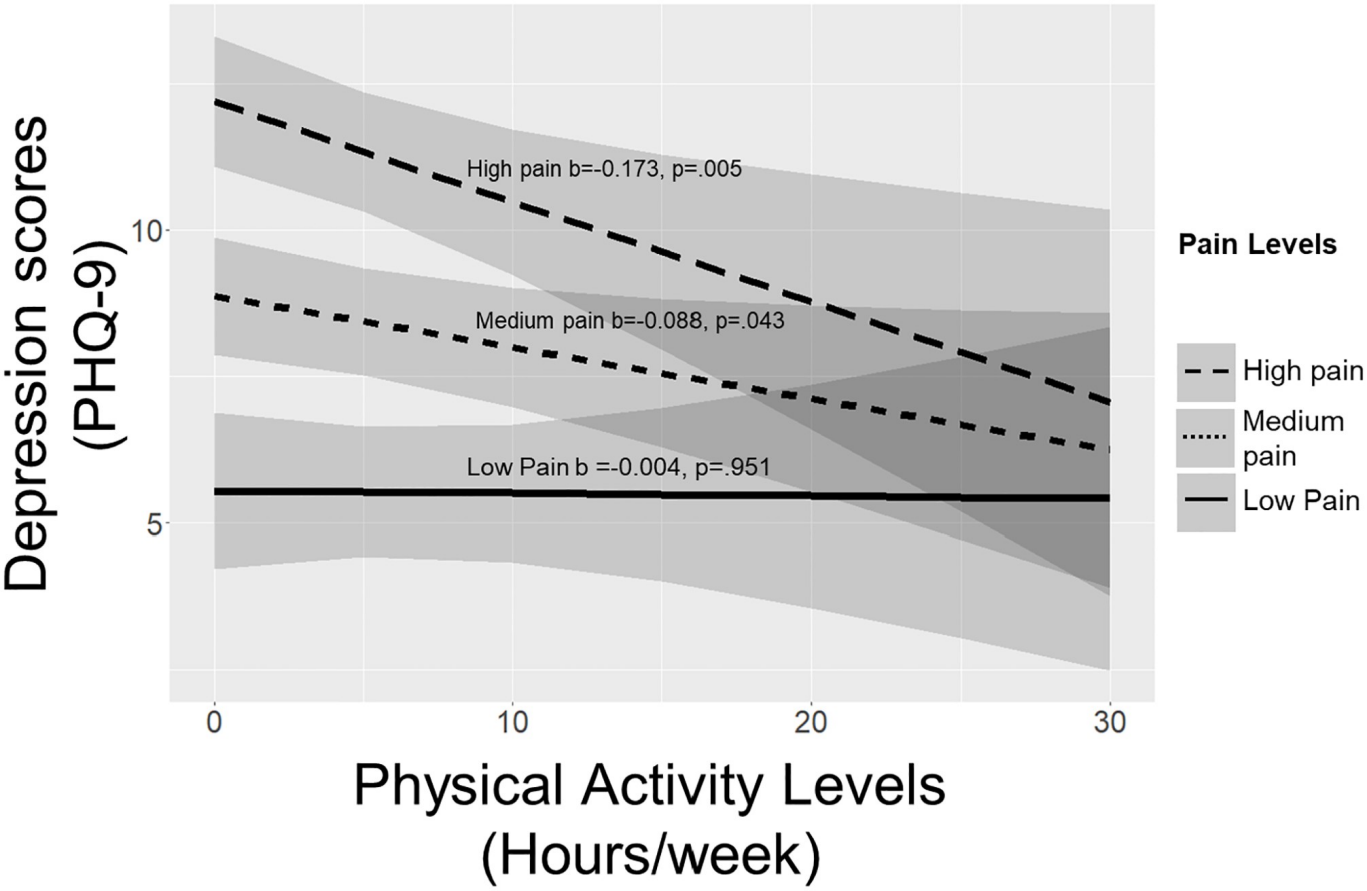
Association between physical activity levels and depression scores in people with low, medium and high pain levels. Low pain is defined as one standard deviation below the mean reported pain levels. Medium pain is defined as the mean reported pain levels and high pain is defined as one standard deviation above the mean reported pain levels. On average, participants reported engaging in 5.19 (SD = 6.5) hours of total physical activity per week.

The moderating effect of pain on the relationship between physical activity and depression are displayed in Table 3. Pain significantly moderated the association between physical activity and depression (p = .043) and the association between physical activity and depression scores was strongest among participants experiencing the highest levels of pain (Fig 1). Simple slope analysis demonstrated that there was a significant inverse association between physical activity and depression when pain was one SD above the mean (b = -0.173, p = .005), and pain levels at the mean (b = -0.088, p = .043), however not when pain was one SD below the mean (b = -0.004, p = .951).

**Table 3:** Moderation analysis examining the moderating effect of pain on the relationship between physical activity and depression.

	B	95%CI	p-value
Sex			
Male	-	-	-
Female	0.43	-0.59, 1.45	.411
Age	-0.08	-0.13, -0.03	.002
Joint Affected			
Knee (ref)	-	-	-
Hip	-1.78	-3.21, -0.35	.015
Shoulder	-0.61	-2.11, 0.89	.424
Foot	0.23	-1.37, 1.83	.775
Ankle	-1.05	-3.15, 1.05	.327
Multiple	-0.55	-3.30, 2.20	.696
Other	-0.14	-3.11, 2.83	.926
Physical Activity	0.18	-0.05, 0.41	.121
Pain	0.39	0.32, 0.46	< .001
Physical Activity * Pain	-0.01	-0.02, -0.00	.036

CI = Confidence interval. Significance was p <0.05.

### Mediation analysis

**Fig 2:**
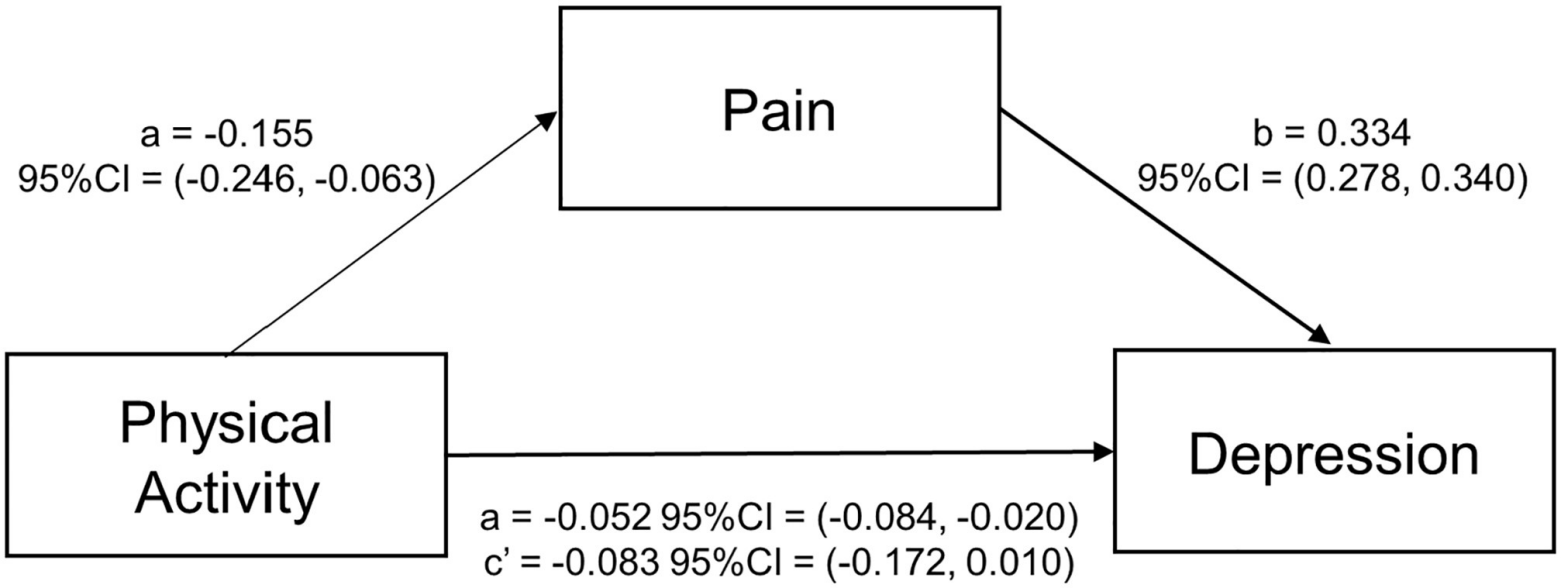
Mediation analysis between physical activity, pain, and depression. CI = Confidence interval. a, b = indirect association between physical activity and depression through pain. c = direct association between physical activity and depression.

Results from the mediation analysis showed that there was a significant indirect association between physical activity and depression through pain (p = .002), and a non-significant direct association (p = .068; Fig 2). This mediation indicates that physical activity is inversely associated with depression symptoms, in part, through lower pain levels associated with participation in physical activity.

## Discussion

In this study it was aimed to understand the impact of pain on the relationship between physical activity and depression symptoms in people with OA. We report that pain significantly moderated the association between physical activity levels and depression, such that the benefits of physical activity on depression was most pronounced in those individuals with the highest level of pain, highlighting the potential efficacy of engaging in physical activity to promote mental wellbeing of individuals with OA, particularly those experiencing intense pain.

Consistent with previous work [5, 6], it was noted that depression symptoms are positively correlated with pain levels [5, 6] and inversely correlated with physical activity levels in people with OA [6, 14, 15]. However, we further highlight the role of pain as a mediator of the association of physical activity with depression symptoms, and that the greatest inverse association between physical activity and depressive symptoms were found in those individuals experiencing the highest levels of pain. This latter finding is important as individuals with moderate to severe levels of pain are overwhelmingly less likely to engage in physical activity due to pain-related barriers [20–22]. Additionally, the severity of the depressive symptoms can influence the mediating effect of pain, with greater severity reducing the direct and indirect effects of pain on physical activity [31]. Thus, interventions prescribed to individuals with OA should involve a multimodal approach that encompasses ample educational support in both physical activity and mental health and informed by effective behaviour change strategies (including via online, self-directed modes as appropriate). Such initiatives should include the benefits of being active, especially in relation to pain reduction and counteracting the beliefs that pain during exercise is to be avoided, given that individuals with OA commonly hold this belief [32–34].

The current study’s findings further highlights that the inverse association between physical activity and depression symptoms is mediated by levels of pain. While understanding the effectiveness of specific types of exercise was outside the scope of the current manuscript, results from a recent network meta-analysis showed that aerobic and mind-body exercise may be highly effective methods of decreasing pain in people with hip and knee OA [35]. Other work and recent guidelines for OA management also demonstrated that resistance exercise may be a highly effective method of decreasing pain in people with hip and knee OA [36, 37].

However, as people affected by OA face a complex array of barriers to physical activity participation [20], and all forms of physical activity are likely to be beneficial for pain in people with OA [35], any form of physical activity or exercise regime that is achievable, enjoyable and sustainable should be encouraged for people affected by OA.

While the holistic benefits of exercise to individuals with OA is not a novel finding in itself, the demonstration that the highest levels of pain were associated with the most potential benefit in terms of reduction in symptoms of depression from engaging in physical activity is of significance. This suggests that those who have the highest pain and are the least likely to engage in physical activity may have the most to gain. From a clinical perspective, a holistic approach to healthcare may be warranted, in which individuals with OA are provided appropriate mental health supports, educative resources on their condition as well as support for a physical activity program. This type of support could be provided for example, through comprehensive, self-directed education platforms for patients for whom self-management is appropriate. For patients with higher level of care needs, psychological treatment can be provided through mental health specialists such as psychologists and exercise programs can be provided through Accredited Exercise Physiologists (AEPs). In Australia, AEPs are University-trained health care professionals with specialised training in the prescription of exercise and physical activity for chronic health conditions, including mental illnesses [38]. In an Australian context, individuals with a chronic health condition, such as OA, can access an individualised treatment plan with an exercise physiologist through a Chronic Disease Management Plan, which enables Medicare rebates for sessions [39]. However, less than 1% of general practitioners (GPs) refer patients with a chronic illness to AEPs, with patients from non-English-speaking backgrounds being referred at less than half the rate of those from English speaking backgrounds [39]. Additionally, some GPs advise their patients to avoid exercise due to their uncertainty of the benefits of physical activity and their concerns that physical activity could cause further injury [34, 40, 41]. To best support the mental health of individuals with OA, it is necessary that health providers not only refer patients to appropriate mental health specialists, but that they are educated about the benefits of physical activity for depression in people with OA, including those from culturally diverse backgrounds, as well. This requires professional development, education and training on the current evidence demonstrating the effectiveness of physical activity interventions and clinical guidance on how to increase the engagement and maintenance of physical activity in patients with OA [42].

This study has some limitations. First, the cross-sectional nature does not permit examination of the long-term relationship between pain, physical activity and depressive symptoms and we are unable to assess causality. There are several potential confounders (e.g. comorbidities, BMI, medication, disease duration) that we have been unable to account for and in future work it would be valuable to explore the impact of these on the interrelationships between physical activity, pain, and mood. Future work should examine the relationship between pain, physical activity and depressive symptoms using longitudinal data and randomised control trials. Further, there have been some inconsistent findings in the research on the relationship between OA and depression [43] and this may be as many complex factors impact an individual’s mood and studies such as the current work have been unable to account for many of these complex and interrelated factors, such as an individual’s social supports and relationships, access to health care provisions, other life stressors, comorbidities, sleep quality, to name a few [43]. Also, individuals were queried about their levels of current pain, rather than their levels of pain over a specified period of time. It is possible that individuals experiencing high levels of pain at the time of assessment may have also been experiencing greater concurrent psychological distress due to the pain. In future work, it would be beneficial to explore if the relationship between pain, physical activity and mental health outcomes are stable over time and how changes in current pain levels may impact the relationship. Further, the use of self-report instruments measuring physical activity could be considered a limitation, due to the possibility of people reporting either over-estimated or under-estimated rates of physical activity engagement. Participants may experience difficulty accurately recalling the frequency and duration of physical activities. Lastly, data was collected during the COVID-19 pandemic, which may have influenced physical activity engagement levels, elevated participants symptoms of depression, and influenced the results of the study.

The strengths of this study are that it: i) included a large sample of people with OA, ii) explored the impacts of both pain and physical activity on depression rather than physical activity or pain alone as in previous studies, iii) was able to demonstrate that gender, age and affected joint did not impact mental health outcomes, indicating that pain and depression impact mental health across diverse populations, iv) utilised the PHQ-9, which is a validated, reliable measure and is considered the gold standard for determining depression symptom severity.

This study has provided evidence that physical activity is particularly associated with depression symptoms in people with greater OA-related pain. Our finding that individuals with highest levels of pain had the biggest reduction in depression symptoms is important. It suggests that individuals with high pain, and who consequently may be the least likely to engage in physical activity may also have the most to gain. This highlights the need for education of both health professionals and patients on the importance of regular physical activity for pain management and mental health and access to appropriate self-management or clinical interventions to increase and maintain engagement in physical activity. The current work indicates the importance of appropriate care plans and approaches to support individuals, who avoid physical activity due to pain, to safely engage in beneficial physical activity.

## Supporting information

S1 Data Data underlying the findings. hrs = hours; Id = identification; k10ts = Kessler; Psychological Distress Scale; Phq9 = Patient Health Questionnaire-9, WEMWBS = Warwick–Edinburgh Mental Wellbeing Scale.
(XLSX)
